# Carrageenan induced phosphorylation of Akt is dependent on neurokinin-1 expressing neurons in the superficial dorsal horn

**DOI:** 10.1186/1744-8069-8-4

**Published:** 2012-01-13

**Authors:** Jeong IL Choi, Fred J Koehrn, Linda S Sorkin

**Affiliations:** 1 Department of Anesthesiology and Pain Medicine. Chonnam National University Medical School and Hospital, Gwangju, Korea; 2 Department of Anesthesiology, University of California, San Diego, La Jolla, CA 92093, USA

**Keywords:** spinal sensitization, receptor trafficking, PI-3K, inflammatory pain, AMPA, P-Akt, GluA1, NK1 receptor

## Abstract

**Background:**

Paw carrageenan induces activation of phosphatidylinositol 3-kinase (PI-3K) and Akt in dorsal horn neurons in addition to induction of pain behavior. Spinal PI-3K activation is also thought to be required for inflammation-induced trafficking of GluA1, AMPA receptor subunits, into plasma membranes from cytosol. Phosphorylation of Akt has a unique time course. It occurs first in the superficial dorsal horn (0.75 h), then soon dissipates and is followed an hour later by Akt phosphorylation in deeper dorsal horn laminae, primarily lamina V. Initially, we wished to determine if Akt phosphorylation in the deeper laminae were dependent on the presence of lamina I, neurokinin receptor bearing projection neurons. As the study progressed, our aims grew to include the question, whether carrageenan-induced GluA1 subunit trafficking was downstream of Akt phosphorylation.

**Results:**

Rats pretreated with spinal saporin conjugated to a stabilized form of substance P had substantial loss of neurons with neurokinin 1 receptors throughout their superficial, but not deep dorsal horns. Animals pre-treated with substance P-saporin exhibited no change in locomotor ability and a small, but significant decrease in carrageenan-induced mechanical allodynia when compared to animals pre-treated with spinal saporin alone. Importantly, carrageenan-induced phosphorylation of Akt was blocked, in the substance P-saporin treated group, throughout the spinal cord grey matter. In marked contrast, carrageenan induced-trafficking of the GluA1 receptor subunit increased equivalently in both treatment groups.

**Conclusions:**

We infer from these data that 1) phosphorylation of Akt in the deep dorsal horn is dependent on prior activation of NK1 receptor bearing cells in superficial dorsal horn, and 2) there are parallel spinal intracellular cascades initiated by the carrageenan injection downstream of PI-3K activation, including one containing Akt and another involving GluA1 trafficking into neuronal plasma membranes that separately lead to enhanced pain behavior. These results imply that the two pathways downstream of PI-3K can be activated separately and therefore should be able to be inhibited independently.

## Background

Akt is a serine/threonine kinase that plays a pivotal role in many essential cellular processes including cell survival and apoptosis. Spinal phosphorylation of neuronal Akt at both the ser 473 and the thr 308 sites occurs following peripheral tissue injury; this can be observed throughout the superficial dorsal horn [1-4], but also is elicited prominently in lateral lamina V and in α-motor neurons [3]. This is a topic of interest, as spinal blockade of Akt phosphorylation (P-Akt) or phosphatidylinositol 3-kinase (PI-3K), its upstream activator, results in amelioration of injury-induced pain behavior [1,2,4,5]. More generally, phosphorylation of Akt is held to be an indicator of neuronal, nociceptor in particular, activation and sensitization [6]. Our recent investigation of this phenomenon using immunohistochemistry for P-Akt (ser473) following carrageenan injection into the plantar hindpaw, revealed an unexpected time course of P-Akt expression throughout the dorsal horn grey matter [3]. At 0.75 h after paw injection, the number of P-Akt positive neurons increased substantially in the superficial dorsal horn compared to basal levels with no change observed in laminae IV or V. In stark contrast, at 2 hours after paw injection, we observed no remaining Akt phosphorylation in the superficial laminae and development of a pronounced increase in P-Akt positive neurons in lamina V. Equally surprising, examination of the ventral horn revealed a small increase in P-Akt in the α-motor neurons at 0.75 h, which had largely dissipated by the 2.0 h time point. These last results are parallel to, and coincident with, results seen in superficial dorsal horn. The present study re-examined this laminar specific time course in the presence and absence of spinal pre-treatment with stabilized substance P-conjugated to saporin (SSP-Sap). This agent, when used at the appropriate dose and duration after administration, is a specific neurotoxin, which kills neurons with neurokinin 1 receptors in the superficial but not the deeper laminae of the dorsal horn [7,8]. The purpose of these experiments was to ascertain if loss of these superficial neurons was sufficient to prevent phosphorylation of Akt induced by paw carrageenan in laminae V and IX. Our initial thought was that activation of NK1 positive projection neurons in superficial dorsal horn [1,9] triggered a bulbospinal pathway, which in turn, activated the deep dorsal horn neurons of lamina V [10,11]. Blockade of P-Akt in motor horn neurons was postulated to occur via local actions of the NK1 positive projection neurons through their axon collaterals [12] or polysynaptically via NK1 positive excitatory interneurons in the superficial dorsal horn [13,14]. Parallel experiments were conducted to determine the effects of SSP-Sap pretreatment (loss of superficial dorsal horn NK1 receptor bearing neurons) on paw carrageenan-induced pain behavior and increases in plasma membrane-associated GluA1 in dorsal horn, an index of increased AMPA receptor trafficking, which is thought to contribute to spinal long- term potentiation and sensitized pain states [15].

In short, our results indicate that loss of NK1 positive neurons in superficial dorsal horn, including presumptive nociceptive projection neurons, substantially blocks carrageenan-induced P-Akt expression in all laminae. Surprisingly, pre-treatment with SSP-Sap had only a modest anti-allodynic effect on enhanced mechanical withdrawal thresholds and no effect on carrageenan-induced GluA1 subunit increases in plasma membrane.

## Results

### NK1 receptor

**Figure 1:**
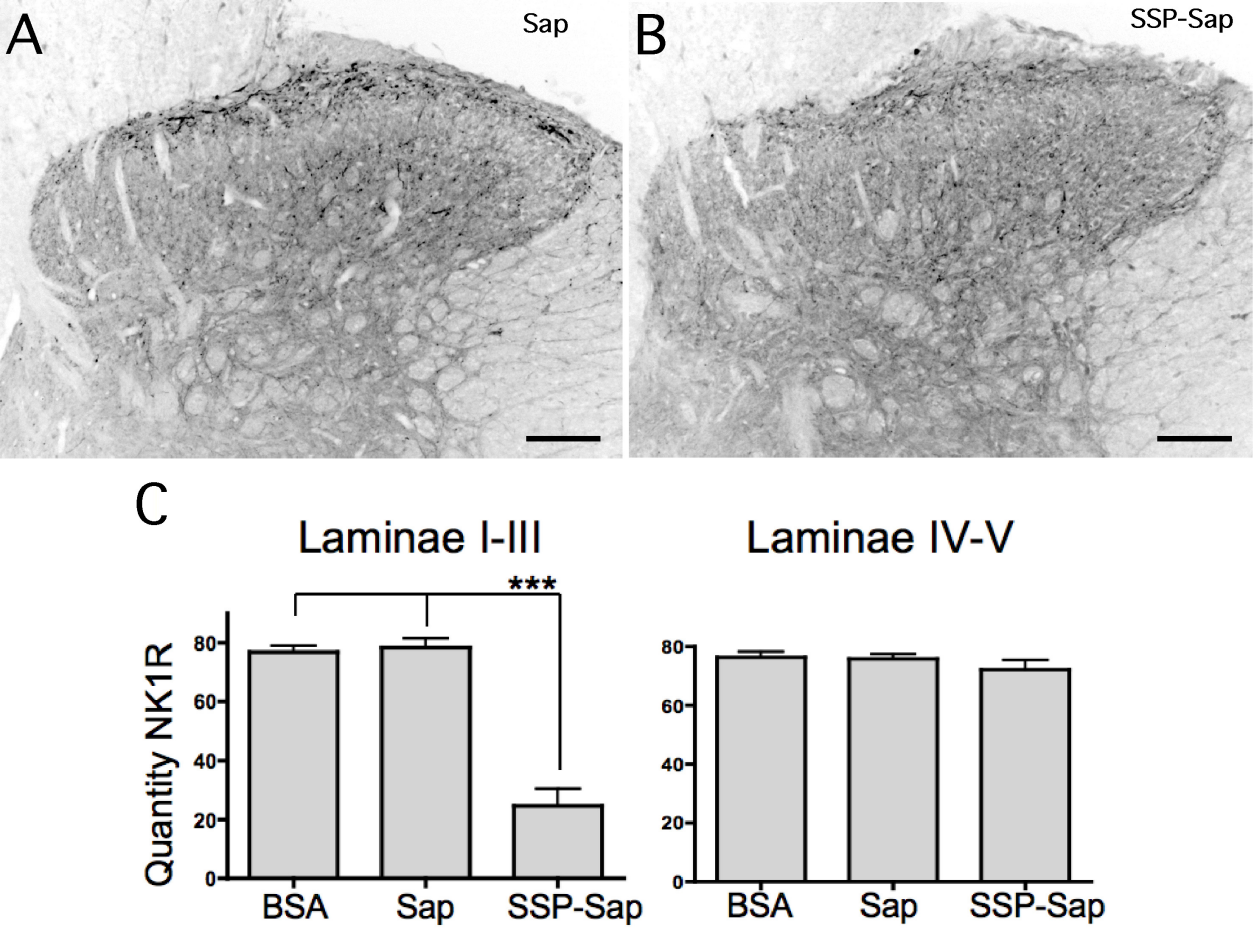
**Micrographs illustrate typical lumbar dorsal horn staining for NK1 receptor in animals pretreated with spinal Sap (A) or SSP-Sap (B) 10 days prior to perfusion**. The majority of the NK1R staining, including staining of almost all large somata, is missing in superficial dorsal horn after the SSP-Sap pretreatment. Size marker = 100 μm. C. Graphs show mean pixel count in laminae I-III and in laminae IV-V following the different spinal pretreatments. *** = p ≤ 0.0001 N = 3 for BSA pretreatment and 8 each for Sap and SSP-Sap.

Otherwise naïve animals pre-treated with BSA (one series only) or Sap (two series of experiments) exhibited NK1 receptor positive structures throughout the superficial dorsal horn as well as in laminae IV and V (Figure 1A), as previously published by others [16,17]. There appeared to be a higher concentration of NK1 staining in the lateral half of the grey matter. This lateral tendency was more pronounced in the deeper laminae. In the ventral horn, NK1 staining was clearly higher in motor neurons than in the surrounding neuropil. This observation was not quantified, however, NK1 receptor staining on motor neurons has been shown previously by Basbaum and several collaborators [16,18]. There were no obvious differences between the BSA and Sap treated animals (Figure 1C). In contrast, ten days after intrathecal SSP-Sap administration, immunohistochemistry revealed depletion of NK1-like receptor in laminae I-III compared to levels observed in Sap or BSA pretreated animals (Figure 1B, C). Levels of pixel intensity in arbitrary units were reduced on average by 68.3%; p ≤ 0.01 (Figure 1C). Few large NK1 receptor positive neurons were seen in laminae I-III and the remaining receptor appeared to be located in small cells or scattered in the neuropil. Levels of NK1 receptor in the deeper dorsal horn laminae showed no significant difference among the three pretreatment groups; there was on average a 5.08% decrease compared to the combination of BSA and Sap pre-treated animals (Figure 1D). NK1 staining intensity on motor neurons appeared to be unchanged in all but one animal, which had developed motor deficits and was eliminated from the study prior to immunohistochemical analysis. There were no obvious differences in NeuN staining among the groups at any laminar level (not shown).

### Behavior

#### Locomotor ability

**Figure 2:**
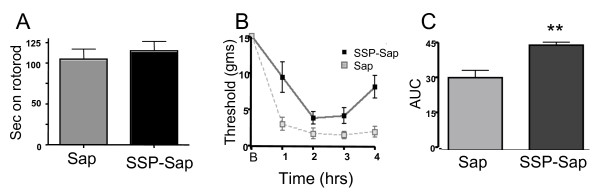
**A. Pretreatment with Sap or SSP-Sap made no difference in time spent on an accelerating rotarod before falling off**. B. Rats pretreated with Sap developed a profound mechanical allodynia following carrageenan as shown by their 50% probability withdrawal threshold to von Frey filaments. Animals pretreated with SSP-Sap also developed hyperalgesia, but thresholds were elevated compared to SAP pretreated animals at all timepoints except at 2 h post-carrageenan. C. Area under the curve for the two treatments indicates that SSP-Sap reduced allodynia integrated over the entire time period. ** = p ≤ 0.001

Animals in the SSP-Sap pretreatment group were indistinguishable from untreated animals in all aspects of their general behavior. Loss of NK1 receptor bearing neurons in the superficial dorsal horn did not result in motor deficits as determined by rotarod testing; p ≤ 0.58. Animals in the Sap pretreatment group had mean times of 104.7 s ± 12.4 on the rotarod before falling off, while the average for animals pretreated with SSP-Sap was 114.7s ± 12.0 (Figure 2A).

#### Mechanical withdrawal threshold

Prior to carrageenan injection, mechanical withdrawal thresholds were the same in Sap (control) and SSP-Sap pretreated rats. These data are consistent with electrophysiological findings reported by Suzuki et al. [10], who found that stimulation of skin with von Frey hairs having 1-15 g bending force produced equivalent numbers of action potentials in lamina V wide dynamic range neurons recorded from both saline and substance P -saporin pretreated animals prior to peripheral tissue insult. Thus, both behavioral (our data) and electrophysiological data from others indicate that responses to mechanical stimulation of the paw in the innocuous range do not significantly differ in the SP- or SSP-Sap pretreated animals from those seen in control animals. Following intraplantar carrageenan, injected paws exhibited extreme inflammation, up to and including the ankle, regardless of intrathecal pretreatment. In rats pretreated with Sap, carrageenan evoked mechanical allodynia was comparable to that seen in previous experiments without pretreatment. Decreases in threshold were prominent within 1 h and remained at this plateau for the remainder of the observation period; p ≤ 0.0001, repeated measures ANOVA (Figure 2B). Thresholds at all time points were significantly less than baseline (p ≤ 0.0001, Bonferroni multiple comparison test). Although mechanical withdrawal thresholds of rats pretreated with SSP-SAP also decreased compared to baseline following paw carrageenan injection (p ≤ 0.0001), the decrease was significantly less than in control Sap animals. The area under the curve for each of the two groups is illustrated in Figure 2C(p ≤ 0.01). Thus, SSP-Sap and the resultant loss of NK1 receptor expressing neurons in superficial dorsal horn diminished, but did not eliminate carrageenan-induced pain behavior.

#### P-Akt

**Figure 3:**
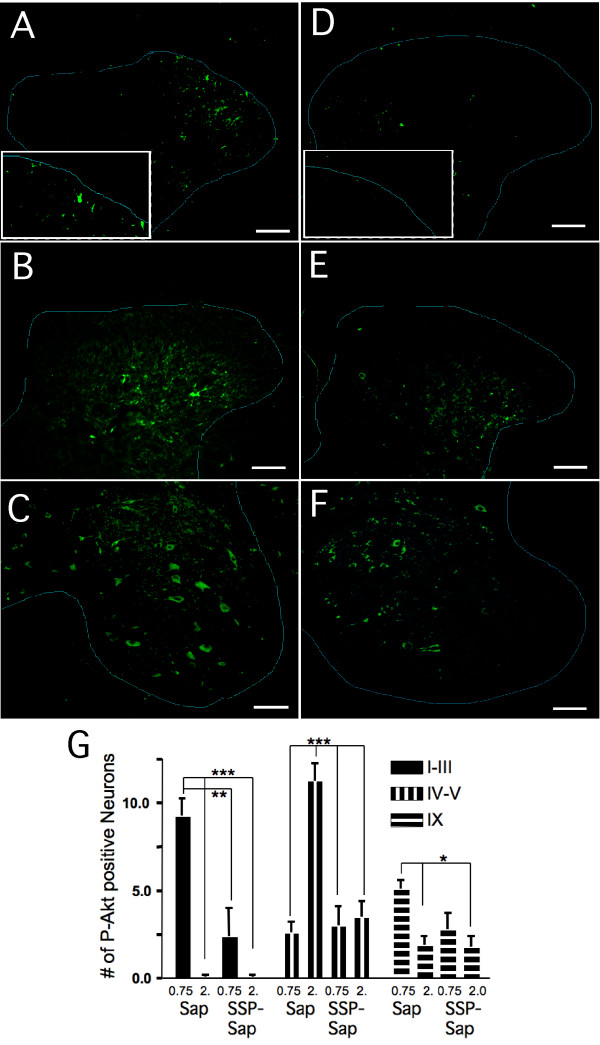
**A-C Spinal cords from Sap pre-treated animals**. A shows dorsal horn from rat perfused 0.75 h post carrageenan, green = P-Akt, most of the P-Akt is in the superficial dorsal horn. Calibration mark = 100 μm and is the same for all dorsal horn pictures. The middle panel (B) shows dorsal horn from an animal perfused 2.0 h post carrageenan. Note that P-Akt activity has shifted from the superficial to the deeper laminae. The bottom panel (C) shows P-Akt in ventral horn 0.75 h post-carrageenan. Calibration mark = 50 μ. Pretreatment with SSP-Sap reduces carrageenan-induced P-Akt in superficial dorsal horn (D) and ventral horn (F) at 0.75 h and in laminae IV and V at 2 h (E). G Mean number of P-Akt positive neurons ± SEM counted in each area under the two conditions, Sap and SSP-Sap pretreatment. Cells were counted only if the P-Akt was co-localized with NeuN (not shown). * p ≤ 0.05, **p ≤ 0.01, *** p ≤ 0.001 Pictures (A-F) and counts (G) are from sides ipsilateral to the injection.

The carrageenan-induced pattern of P-Akt immunoreactivity in BSA (not shown) and Sap pretreated animals was the same as previously reported in untreated animals [3]. Forty-five min after intraplantar carrageenan, we observed the majority of P-Akt positive neurons in the superficial dorsal horn (Figure 3A). All of the sections showed prominent staining in the lateral tissue, although not in extreme lateral tip. There seemed to be more variability in the relative staining intensity of the P-Akt in the medial superficial dorsal horn. At this time, carrageenan also induced P-Akt in a substantial number of motor neurons (Figure 3C), with fewer positive neurons in lamina IV and the lowest density in lamina V (Figure 3A). When tissue was harvested 2 h post carrageenan injection (Figure 3B and 3G), P-Akt positive neurons were rarely seen in the superficial dorsal horn (p ≤ 0.001; compared to 0.75 h) and staining of motor neurons was reduced (p ≤ 0.05; compared to 0.75 h). In contrast, the number of immunopositive neurons in lamina IV-V increased more than 400% (p ≤ 0.001). These time-dependent peaks in carrageenan induced P-Akt immunopositive neurons in superficial dorsal horn and in motor neurons at 0.75 h, and in laminae IV-V at 2 hours were similar to those observed in our previous study with no intrathecal pretreatment and bilateral carrageenan injections [3].

Following pretreatment with SSP-Sap, the number of P-Akt positive neurons observed at 0.75 h in the superficial dorsal horn and at 2 h in laminae IV and V were significantly reduced compared to those seen in the Sap pretreated animals (Figure 3D-G). Although the difference in number of stained motor neurons between the two different pre-treatment paradigms did not reach significance, carrageenan no longer elicited a significant increase in staining (Figure 3G). Under basal conditions, with no carrageenan, we previously observed infrequent P-Akt positive neurons in the superficial dorsal horn and an average of 2-3 P-Akt positive neurons in laminae IV-V; thus, SSP-Sap pretreatment prevented all of the carrageenan-induced Akt activation in the deep dorsal horn and virtually all of that seen in superficial dorsal horn.

#### Membrane GluA1

**Figure 4:**
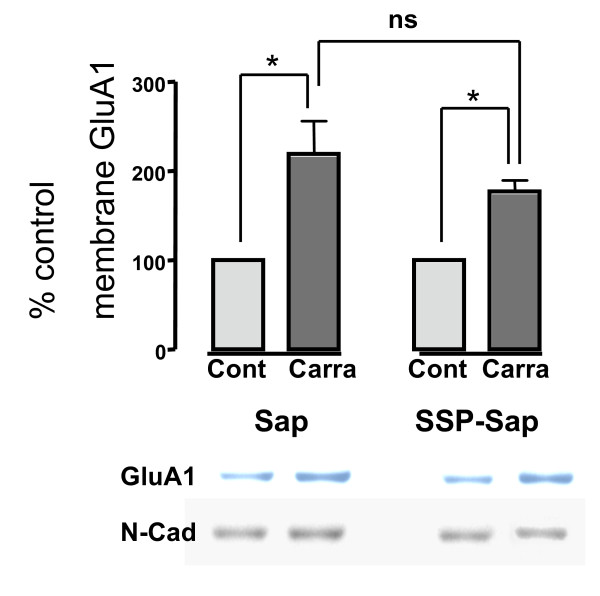
**In both Sap and SSP-Sap pretreated animals, intraplantar carrageenan elicited a doubling of GluA1 subunits in the crude plasma membrane fraction**. There was no difference in the percent increase between the treatments. Representative blots for GluA1 are shown for each condition. Ns = non-significant.

As we have seen previously in naïve animals, paw carrageenan in control Sap pretreated animals, elicited approximately a doubling of plasma membrane GluA1; p ≤ 0.05, N = 3-5 (Figure 4). In SSP-Sap pretreated animals, carrageenan also elicited an increase in plasma membrane GluA1; p ≤ 0.05, N = 3-5. This percent increase in membrane GluA1 was no different than that observed in the Sap controls; p > 0.05. Given that the SSP-Sap animals had substantially less P-Akt, these data suggest that paw carrageenan-induced trafficking into the membrane is Akt independent.

## Discussion

**Figure 5:**
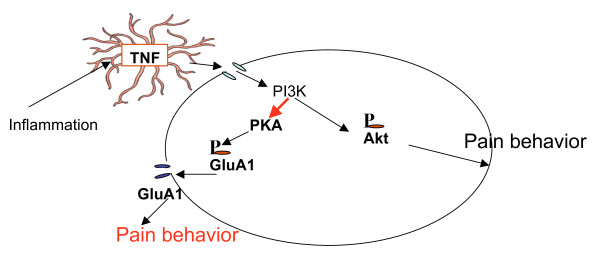
**Schematic of hypothesis: Inflammation induced release of spinal TNF activates PI-3K probably via TNF receptor 1**. PI-3K leads to phosphorylation of Akt, both directly and indirectly through PDK1. P-Akt activates signal transduction cascades leading to central sensitization. PDK1 also activates PKA, which phosphorylates GluA1 at ser 845. Phosphorylation at this site lowers the energy required for GluA1 insertion into the plasma membrane as part of a functional AMPA receptor. Increased AMPA receptor density, especially AMPA receptors enriched with GluA1 rather than GluA2, contributes to spinal long term potentiation and pain behavior. Despite the fact that they both are initiated through a PI-3K linkage, phosphorylation of Akt and GluA1 trafficking seem to be independent of one another.

The most striking findings of this study are that pre-treatment with SSP-Sap: 1) blocked the carrageenan-induced expression of P-Akt throughout the dorsal horn as well as in α-motor neurons; 2) significantly reduced, but did not eliminate carrageenan-induced mechanical allodynia and 3) did not decrease carrageenan-induced increases of GluA1 trafficking into the membrane. Taken together we infer from these data that 1) phosphorylation of Akt in the deep dorsal horn is dependent on prior activation of NK1 receptor bearing cells in the superficial dorsal horn, 2) there are parallel spinal intracellular cascades initiated by the carrageenan injection, including one containing P-Akt, that separately lead to enhanced pain behavior, and 3) carrageenan-induced GluA1 trafficking into the plasma membrane and the pain behavior resulting from this process are not downstream of P-Akt (Figure 5).

Immunohistochemical data indicate that the SSP-Sap treatment was successful in substantially reducing NK1 staining in the superficial dorsal horn. Lamina I neuronal populations with high densities of NK1 receptor include approximately 80 percent or more of all spinoreticular, spinoparabrachial and spinothalamic tract neurons [9,19-21]. It is unlikely that there was significant loss of NK1 receptor expressing neurons in deeper laminae. Although Nichols et al. demonstrated measurable loss of lamina III neurons at 30 days after SP-Sap and throughout the dorsal horn at 100 and 200 days post infusion of the toxin [22]; Wiley et al., using the SSP-Sap at the same dose and survival time (seven days) that we employed, demonstrated a selective loss of lamina I neurons with no neuronal decrease in laminae III-VI or × [7]. An exciting recent paper from Todd's group describes two populations of NK1 receptor bearing projection neurons, larger neurons with GluA4 AMPA receptor subunits and medium sized neurons that were positive for GluA1, rather than GluA4 [23]. They also identified a third population of smaller neurons, having lower NK1 receptor densities, that were not projection cells [21]. Neurons of this class, which numerically is the largest of the three, are not GABAergic in lamina I [24], thus a large number of them are presumptive excitatory interneurons. Al Ghamdi and colleagues have posited that due to their low level of receptor, this interneuronal population may not be killed by spinal saporin linked to substance P. This hypothesis suggests that the roughly 30% of the NK1 immunoreactivity, as determined by pixel count, that remained in the superficial dorsal horn after SSP-Sap pretreatment could be attributed to retention of this interneuronal population. Importantly, loss of NK1 receptor bearing neurons in the superficial dorsal horn prevented not only carrageenan-induced P-Akt in superficial dorsal horn, but also blocked induction of P-Akt in the deeper dorsal horn lamina and reduced it in the motor neurons. Since development of P-Akt in superficial dorsal horn and motor neurons has the same time course, we originally thought that loss of presumptive motor neuron sensitization was due to loss of excitatory interneurons in the superficial dorsal horn that are part of the flexion reflex arc. However, if, as hypothesized above, those neurons are retained, the carrageenan-induced induction of motor neuron P-Akt must be due to a different mechanism. The motor neuron response could be triggered via local collaterals of the NK1 receptor bearing projection neurons [12](acting as a first step); alternatively, the motor neurons could be activated by a spino-bulbo-spinal loop, which uses the projection neuron as the ascending leg. Our data does not allow us to decide between these alternatives. The pronounced time difference observed between appearance of P-Akt in superficial and deeper dorsal horn laminae could be due to involvement of different signal transduction cascades in different neuronal populations. These neurons could possess different time courses, leading to the same endpoint (P-Akt). Alternatively, induced P-Akt in deeper laminae could be due to a complex spino-bulbo-spinal pathway, which has been observed by others to be involved in peripheral tissue or nerve injury-induced sensitization of deep dorsal horn neurons [20,22,25,26] or to some combination of the two. Further experiments will have to be undertaken to delineate the difference in induction time.

Our rotarod data indicate that loss of projection neurons in the superficial laminae does not decrease locomotor activity, thus local proprioceptive circuitry appears to be intact. This is consistent with the reported lack of change in overall behavior [22] and maintained flinching frequency in the phase 1 formalin test [10] following spinal administration of substance P-Sap.

The overall decrease of enhanced pain behavior seen in Figure 2B was modest compared to the loss of spinal sensitization seen following similar SP-Sap pre-treatment prior to intradermal capsaicin, which substantially eliminates mechanical and thermal hyperalgesia after capsaicin injection into the paw [8,27]. Phase 2 activity of the formalin test is also substantially reduced by both SP-Sap and SSP-Sap pretreatments [7,10,22].

However, at several timepoints following injection of complete Freund's adjuvant (CFA) [10] and carrageenan [22], these same investigators observed a substance P-saporin associated attenuation rather than obliteration of inflammation-induced mechanical allodynia that was more similar to our results. Differing degrees of attenuation may represent differences in sensitizing mechanisms, perhaps involving multiple redundant pathways between the more slowly developing, longer lasting inflammatory models such as carrageenan and CFA vs. capsaicin or formalin injection, which produce a more brief barrage of afferent drive. Other reasons for greater residual allodynia in our study may include greater magnitude of allodynia in our control animals or variations in testing protocols. The greatest loss of pain behavior was seen at the first hour post-carrageenan when the model was in transition between pain behavior due to trauma secondary to the injection and pain behavior secondary to peripheral inflammatory processes [28].

In marked contrast to the significant residual carrageenan-induced mechanical allodynia seen after pretreatment with the SSP-Sap, our data indicate that the pathway leading to Akt activation was profoundly suppressed by the chemical lesion in the superficial dorsal horn. Akt is a second messenger downstream of phosphatidylinositol 3-kinase (PI-3K) and mediates many of its survival functions through phosphorylation and regulation of transcription factors including nuclear-factor- kappa B, mTor, various caspases and GSK3. Antagonism of the PI3K/Akt pathway at the level of the spinal cord and dorsal root ganglia is thought to be anti-hyperalgesic. Spinal pretreatment with various Akt inhibitors attenuates the phase 2 formalin response [1] and thermal hyperalgesia and mechanical allodynia resulting from spinal nerve ligation [4]. Willis's group has shown that Akt inhibition results in total blockade of mechanical hyperalgesia induced by intradermal capsaicin [2], again pointing to potential differences in magnitude between acute capsaicin responses and longer term inflammatory conditions. Importantly, our group has reported only attenuation of carrageenan-induced allodynia in animals pretreated with spinal Akt inhibitors [1,3]. However, despite these data implicating Akt in spinal sensitization, a recent study demonstrated that morphine-mediated peripheral analgesia is Akt mediated [29].

Prior to insertion into the plasma membrane, GluA1 subunits of AMPA receptors, contained within cytosolic endosoms, are phosphorylated by PKA at ser 845. In the hippocampus, this process appears to be necessary, but not sufficient for extrasynaptic membrane insertion of GluA1 containing AMPA receptors [30-32]. Other studies have shown that spinal inhibition of PKA blocks GluA1 trafficking in the dorsal horn following capsaicin injections [33]. Our previous work indicates that spinal pretreatment with the TNF antagonist etanercept blocks carrageenan-induced increases in P-Akt, P-GluA1 ser845 and trafficking of GluA1 into the plasma membrane [3]. We hypothesized at the time 1) that TNF acting through one of its receptors led to activation of PI3K and Akt and 2) that PKA phosphorylation of GluA1ser845 and trafficking into the membrane was downstream not only of the spinal TNF release, but also of the PI3K and Akt activation. However, this hypothesis requires that activation of PKA is downstream of P-Akt. We have not seen clear proof of this linkage in the literature. However, we have seen evidence that PDK1, which is upstream of P- Akt can activate or prime PKA, as well as PKC [34-36]. Thus, in light of our present data we believe that GluA1 receptor trafficking is independent of Akt (Figure 5).

Using neuronal tracing and post-embedding immunogold labeling to examine primary afferent fiber synapses in the superficial dorsal horn, Larsson and Broman [37] have shown that following noxious stimulation, the majority of AMPA receptors with enriched GluA1 subunit composition were post-synaptic to non-peptidergic primary afferent C fibers with a far less prominent increase in GluA1 postsynaptic to the peptidergic primary afferent fibers. This is in keeping with the lack of correspondence that we observed between loss of NK1 receptor-bearing neurons and increased translocation of GluA1 at the plasma membrane. It also brings up the possibility that rather than two parallel signal transduction cascades in the same neurons, different populations of neurons exhibit Akt phosphorylation and GluA1 trafficking in response to intraplantar carrageenan.

## Conclusions

We infer from these data that 1) phosphorylation of Akt in the deep dorsal horn is dependent on prior activation of NK1 receptor bearing cells in lamina I, 2) there are parallel spinal intracellular cascades or separate neuron pathways initiated by the carrageenan injection. One includes Akt phosphorylation and a second is Akt independent and ends in GluA1 movement into plasma membranes.

## Methods

### Animals

Male Holtzman rats (Harlan Industries, Indianapolis, IN, USA) weighing 250-300 g were housed in pairs on a 12-h light/dark cycle with controlled temperature and free access to food and water. Efforts were made to minimize animal discomfort and reduce numbers of animals used. All experiments were carried out according to the National Institute of Health Guide for the Care and Use of Laboratory Animals, and the Institutional Animal Care and Use Committee of the University of California, San Diego approved this study protocol.

In preparation for the actual experiments, rats were anesthetized with isoflurane (4% for induction, 2% for maintenance) and a polyethylene-5 (PE5) catheter (Scientific Commodities, Inc., Lake Havasu City, AZ) was inserted into the intrathecal space caudally from the cisterna magna and ending over the thoraco-lumbar junction. Saporin or 100 ng of [Sar9Met(O2)11] substance P coupled to saporin (SSP-Sap, Advanced Targeting Systems, San Diego, CA) was injected through the catheter in a 10 μl volume, followed by 10 μl of saline, and the catheter then remained in place for 30 min before its removal. The addition of Sar9Met(O2)11 to substance P conjugated to saporin makes the agent more stable and potent than substance P alone bound to saporin. The dose, injection volume and pre-treatment interval that we used were based on Wiley et al., who observed no loss of lumbar dorsal horn neurons expressing the NK1 receptor in deeper laminae and prominent loss of NK1 receptor in lamina I [7]. In one set of control animals intended for histological analysis, our first pre-treatment injection was 10 μl bovine serum albumin (BSA) rather than Sap or SSP-Sap.

The skin incision overlying the dural entry site was closed with 3-0 silk suture and animals were allowed to recover in their home cages for 10-14 days. Rats received a 5 mL subcutaneous injection of Lactated Ringer's solution (Baxter HealthCare Corporation, Deerfield, IL, USA) containing carprofen (5 mg/kg; Pfizer Animal Health, New York, NY, USA) for potential pain relief immediately after surgery and again on the following day. After recovery from anesthesia, any rats with motor or postural deficits (less than 5%) were immediately sacrificed by inhalation of carbon dioxide followed by bilateral pneumothorax. Interestingly, one animal developed motor weakness seven days post injection; the animal was perfused at this time and lumbar spinal cord processed for NK1-like immunoreactivity. Histological analysis showed a prominent loss of NK1 staining in the ventral horn (data not shown). Data from this animal were not included in any analysis.

### Carrageenan-induced inflammation

Carrageenan (degraded λ-Carrageenan, Wako Pure Chemical Industries, Japan) was dissolved in saline to form a 2% solution and stored at room temperature for 24 h; 100 μl of this solution was injected subcutaneously into the center of the ventral surface of the left hind paw under light isoflurane anesthesia using a 30 g needle. Carrageenan injection was unilateral.

### Behavioral testing

#### Locomotor testing

Animals were trained on an accelerating rotarod (Columbus Instruments, Columbus, OH, USA). Training consisted of two or more 1 min trials at 4 rpm on each of two sequential days. Once animals would stay on the device for 60 s, they had two sessions with the rod accelerating at 0.1 rpm/s. On day three, animals were placed on the rod for several seconds at 4 rpm before acceleration began. The average of three measures (30 min or more apart) was taken; animals that did not fall off or jump were taken off of the rod 180 s after the acceleration began. The person performing the behavioral testing was blinded as to the chemical nature (Sap or SSP-Sap) of the pre-treatment.

#### Mechanical Threshold

Animals were acclimated to the testing room and apparatus (one hour in their home cage and 1 hour in the test chamber) on three separate days prior to data collection. On the day of the experiment, rats were brought to the testing room and left in their cages for at least 30 min and then placed in individual Plexiglas test chambers with wire mesh floors for another 30 min prior to testing. Mechanical withdrawal thresholds were assessed with a set of von Frey filaments (Stoelting, Wood Dale, IL, USA) having buckling forces between 0.41 and 15.2 g. The paradigm was based on the up-down test [38] to obtain the 50% probability withdrawal threshold. Filaments were applied perpendicularly to the plantar surface of the hindpaw through the wire mesh floor until the filament was just slightly bent. Each application was maintained for 6 seconds or until the animal rapidly lifted or licked the hind paw; both paws were tested. Any rat with a mean or left paw basal withdrawal threshold below 10 g was excluded from the study. After carrageenan injection into the area on the left paw, which had been tested with the von Frey filaments, withdrawal thresholds were re-determined at 1-hour intervals for a 4-hour period. The person performing the behavioral testing was blinded as to the chemical nature (Sap or SSP-Sap) of the pre-treatment.

#### Immunohistochemistry

At specified time points following carrageenan injection, rats were anesthetized with isoflurane and transcardially perfused with cold heparinized 0.9% saline containing phosphatase inhibitors (Sigma) followed by chilled 4% paraformaldahyde in 0.1 M phosphate buffer. Spinal cords were removed and post-fixed in perfusate for 6 h and transferred, first to 20% sucrose for 12-24 hs and then to 30% sucrose for cryoprotection. Tissue was kept at 4°C. The fixed lumbar enlargements were embedded in O.C.T. compound (Tissue-Tek, Torrance, CA, USA) snap frozen, and transverse sections (20 μm) from L4-L5 were cut on a Leica CM 1800 cryostat. Sections were mounted on Superfrost Plus glass slides (Fisher Scientific, Pittsburgh, PA, USA) and double labeled with rabbit monoclonal anti-P-Akt ser 473 (1:200; Cell Signalling, Danvers, MA, USA) or polyclonal anti-NK1 (1:3,000; Advanced Targeting Systems, San Diego, CA, USA) and the cell marker, mouse anti-Neu N (neurons, 1:500; Millipore, Temecula, CA, USA). Reported results were observed in 8 animals each following pretreatment with Sap and SSP-Sap and in 3 animals with BSA pre-treatment; clearly P-Akt immunopositive neurons were counted, under blinded conditions, within the boundaries of laminae I-III, lamina IV-V and the ventral horn. Cells were counted only if there was a clearly visible nucleus and double labeling with NeuN. Ventral horn cells were only counted if they had minimum somal diameters of 25 μm and, thus, were presumptive α-motor neurons. Binding sites were visualized with species matched goat anti-rabbit secondary antibody conjugated with Alexa Fluor 488 (1:500; Invitrogen, Carlsbad, CA, USA) or goat anti-mouse antibody conjugated with Alexa Fluor 594 (1:500, Invitrogen). Equivalent dilutions of normal rabbit or mouse IgG were substituted for primary antibodies as a control for non-specific staining. Images were captured with a fluorescence microscope (Olympus, Melville, NY, USA) at 10-60× with an attached Olympus America digital camera linked to a computer. Single channel fluorescent images were acquired using Image-Pro Plus software (Media Cybernetics, Bethesda, MD USA). NK1 receptor staining was quantified as density in standardized boxes centered in central lamina I-II and lateral lamina V using the saturated fluorescent images. Box placement was performed by an investigator who was unaware of the agent used for pre-treatment. For presentation, images were converted to grayscale, and inverted in pixel intensity (i.e., white to black; with dark pixels representing positive labeling; Figure 1). Multi-channel green-red fluorescent images were acquired and merged to count neurons that were positive for P-Akt labeling. The red NeuN channel was eliminated for the final image (Figure 3).

#### Subcellular membrane fractionation and Western Blots

Crude plasma membrane fraction GluR1 was measured in whole cell homogenates obtained 1 h after paw injection with carrageenan. Animals were deeply anesthestized with isoflurane (5%), decapitated and the spinal cord was extruded with cold saline. After dissecting a 1 cm length of lumbar enlargement (L2-L5), the dorsal quadrant ipsilateral to the carrageenan injection was harvested and immediately frozen with dry ice and stored at -70°C. Frozen tissue was placed in a dounce homogenizer in 3 ml of hypotonic buffer containing protease and phosphatase inhibitors (Sigma, St. Louis, MO, USA**)**, 1 mM NaHCO3 in H2O and homogenized. The homogenate was allowed to rest on ice for 10 min and was then centrifuged at 12,000 rcf for 10 min at 4°C, and the supernatant removed. This (S1) was then centrifuged at 21,600 rcf for 30 min at 4°C. The resulting supernatant (S2) was then subjected to 150,000 rpm at 4°C for 2 h and the resulting pellet containing the plasma membranes was washed in 4 ml hypotonic buffer and centrifuged again at 150,000 rcf for 2 hr. The final pellet was resuspended in 50 μl standard extraction buffer (50 mM Tris, pH 7.4; 150 mM NaCl; 1 mM EDTA, pH 8; 0.5% Triton X-100 with protease and phosphatase inhibitors).

Protein concentration of this final suspension was determined using a bicinchoninic acid (BCA) kit (Pierce Biotechnology Inc., Rockford, IL, USA). Equivalent amounts (15 μg) of protein from each sample were loaded into a Nu-PAGE 4-12% Bis-Tris Gel (Invitrogen, Carlsbad, CA, USA) in MOPS running buffer and transferred onto a nitrocellulose membrane. The membrane was blocked with 5% nonfat milk in Tris-HCl buffer containing 0.1% Tween 20, pH 7.4 (TBS-T) for 1 hour at room temperature and then incubated overnight at 4°C with rabbit anti-GluA1 (1:1000; Millipore, Temecula, CA). The membrane was washed with TBS-T and then incubated with goat anti-rabbit HRP (horseradish peroxidase)-linked secondary antibody (Cell Signalling) for 1 hour on the next day. After incubation the membrane was exposed to SuperSignal West Femto substrate (Pierce Biotechnology, Inc.) to enhance the signal. Following exposure to X-ray film, membranes were stripped and reprocessed for N-cadherin, a marker for plasma membranes **(**rabbit anti-N-cadherin,1:1,000; Cell Signalling) as a loading control. Immunoblots were scanned and densitometric analysis performed using ImageQuant (Amersham Biosciences, Piscataway, NJ, USA). Immunoblot density was normalized to controls run on the same gel.

#### Statistical Analysis

Behavioral data were expressed as mean ± S.E.M. An unpaired t-test was used to compare rotarod duration between the Sap and the SSP-Sap animals. Mechanical withdrawal threshold was also expressed as area beneath the curve and also compared using unpaired t-tests. Withdrawal thresholds from each pretreatment were analyzed using an ANOVA for repeated measures. Membrane fraction GluA1 from carrageenan-injected animals was normalized to the mean of the appropriate non-carrageenan injected samples and compared to carrageenan-injected animals using an unpaired t-test. Cell counts and pixel density were compared using unpaired t-tests. The percentage loss of NK1 staining was calculated by taking the staining intensity in the SSP-Sap animals divided by that of the control animals and multiplying by 100. That gave us the % remaining staining density. To obtain the % decrease, we subtracted this number from 100.

## Abbreviations

AMPA: α-amino-3-hydroxy-5-methyl-4-isoxazolepropionic acid receptor; GABA: γ-Aminobutyric acid; CFA: complete Freund's adjuvant; HRP: horseradish peroxidase; NK1: neurokinin 1; NK1R: neurokinin 1 receptor; PI-3K: phosphatidylinositol 3-kinase; SSP: [Sar9Met(O2)11]; SSP-SP: [Sar9Met(O2)11] substance P coupled to saporin; SEM: standard error of the mean; SP: substance; SP-Sap: substance P saporin.

## Competing interests

The authors declare that they have no competing interests.

## Authors' contributions

JIC performed experiments, assisted in the design, statistical analysis and the writing. FJK performed the immunohistochemistry, tissue fractionation and associated analyses. LSS performed experiments, contributed to the design, data analysis and writing of the manuscript. All of the authors have read and approved the final manuscript.
